# A retrospective study of arthroscopic treatment for patients with bordline developmental dysplasia of the hip

**DOI:** 10.1007/s00264-024-06300-7

**Published:** 2024-09-09

**Authors:** Yu Gou, Zi Zhang, Binyang Meng, Jiangang Cao, Jiawang Zhu, Hongzhou Li, Qian Zhao

**Affiliations:** 1 Department of Orthopaedic Surgery, Tianjin Hospital, Tianjin University, Tianjin, China; 2 Graduate School of Tianjin University, Tianjin University, Tianjin, China

**Keywords:** Arthroscopy, Bordline Developmental Dysplasia of the hip, Hip, Review

## Abstract

**Purpose:**

Hip arthroscopy is effective in treating bordline developmental dysplasia of the hip (BDDH), but there are only a few clinical reports in China, and its postoperative failure rate is still a problem that cannot be ignored. The aim of this study was to analyze the clinical effect of hip arthroscopy in BDDH treatment in China and to explore the risk factors influencing the efficacy of hip arthroscopy in BDDH treatment.

**Methods:**

All of 22 cases of BDDH treated with arthroscopy in our hospital from November March 2017 to February 2022 were analyzed retrospectively, including ten males and 12 females, with an average age of 34.7 ± 9.5 years (19–53 years). All patients underwent arthroscopic treatment with acetabular plasty, labral repair, femoral osteoplasty, and capsular plication. Visual Analogue Scale (VAS), modified Harris Hip Scores (mHHS), Hip Outcome Score—activities of Daily Living (HOS-ADL) and International Hip Outcome Tool-12 (iHOT-12) were measured before operation and at the follow-up, and statistical analysis was performed. The Minimum clinically significant difference (MCID) and Patient Acceptable Symptom State (PASS) were also obtained.

**Results:**

22 patients were followed up, and the follow-up time was ≥ one year, with an average of 21.4 ± 8.2 months. The VAS score decreased from 5.27 ± 1.58 points before surgery to 1.96 ± 0.92 points at the follow-up, and the difference was statistically significant (*t = 9.05*,*P < 0.001*). The mHHS score increased from 64.84 ± 13.58 points before surgery to 90.4 ± 10.11 points at the follow-up, and the difference was statistically significant (*t*=-7.07, *P* < 0.001). The HOS-ADL score increased from 68.92 ± 11.76 points before surgery to 88.91 ± 9.51 points at the follow-up, and the difference was statistically significant (*t=-8.15*,*P < 0.001*). The iHOT-12 score increased from 49.32 ± 12.01 points before surgery to 79.61 ± 15.89 points at the follow-up, and the difference was statistically significant (*t=-7.66*,*P < 0.001*). The MCID (mHHS) and MCID (HOS-ADL) were 81.8% and 77.3% respectively, and the PASS (mHHS) and PASS (HOS-ADL) were 86.4% and 72.7% respectively at the follow-up.

**Conclusion:**

Hip arthroscopy can achieve good short-term outcomes in the treatment of BDDH.

**Level of evidence:**

IV Therapeutic Study.

## Introduction

Adult borderline developmental dysplasia of the hip (BDDH) represents a distinct condition that blurs the boundaries between developmental dysplasia of the hip (DDH) and a normal hip. This condition is primarily characterized by mild acetabular undercoverage, quantitatively assessed through the lateral centre-edge angle (LCEA). LCEA, first proposed by Wiberg in 1939, is used to evaluate the coverage of the femoral head by the acetabulum: A LCEA > 25° denotes a normal hip configuration, whereas a value < 25° indicates DDH. LCEA readings between 20° and 25° are classified as undetermined, thereby defining the threshold for BDDH. Previous studies showed controversy in the definition of BDDH: some studies defined LCEA of 18°-25° as BDDH [1–5], while others defined LCEA of 20°-25° as BDDH [6–8].

BDDH increases the risk of hip osteoarthritis (OA) [9]. Some studies indicated that the risk of hip OA in patients with BDDH is 1.4-2 times higher compared to the normal population [9]. In individuals with an LCEA of < 28°, the risk of radiographic hip OA increases by 13.0% with each 1° reduction in LCEA, while the likelihood of requiring total hip arthroplasty (THA) escalates by 18% [10]. However, this elevated risk is comparatively low among patients with typical DDH [9]. In some patients with BDDH, chronic hip instability leads to articular surface overload, inducing hip OA and causing irreversible damage to the articular cartilage and labrum, thus reducing the life span of the hip joint [11, 12]. Additionally, many BDDH patients may suffer from femoroacetabular impingement due to proximal femoral cam deformity in the hip joint, increasing the risk of hip OA [5, 9, 13]. Surgical treatment is possible for patients with BDDH with poor response to conservative treatment. Currently, hip arthroscopy and periacetabular osteotomy (PAO) are commonly used surgical methods [14, 15].

The surgical treatment of BDDH remains controversial, and the choice of hip arthroscopy over PAO remains unclear [16]. PAO is the gold standard treatment for non-OA hip joints with severe dysplasia [17]. Wyles et al. [18] indicated that PAO effectively changed the natural course of DDH. McClincy et al. [19] followed up and found that 94% of BDDH patients who underwent PAO had significant postoperative effects, with significantly improve mobility, quality of life, and overall health. However, PAO is more invasive compared with hip arthroscopy [16], and minimally invasive surgery is more acceptable to patients. Numerous studies indicated that hip arthroscopy is effective in managing BDDH, demonstrating beneficial outcomes in the short, medium, and long term [2, 20–25]. However, treatment failure rates, ranging from 0 to 46%, and the average reoperation rate of 8.5% for BDDH cannot be overlooked [1, 7, 8, 26]. Currently, domestic research on the use of hip arthroscopy for BDDH remains limited, with only a few clinical reports [27–32]. Consequently, ongoing research is essential to further evaluate the efficacy and safety of this therapeutic approach.

Therefore, this study retrospectively analyzed and studied the clinical data of patients who underwent hip arthroscopy from November 2017 to February 2022. Our research purposes were to (1) Analyze the clinical effect of hip arthroscopy in BDDH treatment and (2) Explore the risk factors influencing the efficacy of hip arthroscopy in BDDH treatment.

## Methods

### Study subjects

In this study, we adopted the consensus recommendations of the Expert Consensus on Diagnosis and Treatment of Borderline Developmental Dysplasia of the Hip (2022) [14]: an LCEA angle between 20° and 25° is defined as BDDH. Adult patients with BDDH underwent hip arthroscopy. The inclusion criteria were LCEA between 20° and 25° and OA Tönnis grade ≤ 1. The exclusion criteria comprised a previous history of other hip joint diseases (fractures, osteonecrosis, etc.), a previous history of ipsilateral hip surgery, narrow hip joint space (Tönnis classification > 1), LCEA < 20°, or > 25 °, follow-up <  one year, and age is < 18 years.

### Baseline characteristics

We retrospectively analyzed clinical data of BDDH patients who underwent hip arthroscopy from November 2017 to February 2022. According to the above-mentioned inclusion and exclusion criteria, 24 cases met the inclusion criteria, of which 22 cases (22 hips) completed the follow-up and were included in this study. The cohort comprised ten males and 12 females, with an average age of 34.7 years (range: 19–53 years) and a mean body mass index (BMI) of 22.7 kg/m^2^. Diagnostic imaging before surgery included pelvic anteroposterior radiographs, Dunn radiographs, and three-dimensional computed tomography (CT) reconstructions of the hip joint, alongside magnetic resonance imaging (MRI) to assess the labrum, cartilage, and any impingement in the affected hip. Measurements of the LCEA and Tönnis angle were conducted using anteroposterior pelvic radiographs, and the α angle was measured using Dunn radiographs. Regarding the measurement details for the pelvic anterior-posterior X-ray, the patient was in the supine position with the legs 15º internally rotated to compensate for femoral antetorsion and to provide better visualization of the contour of the lateral femoral head-neck junction. The film-focus distance was 1.2 m, and the central beam was directed to the midpoint between a line connecting both anterosuperior iliac spines and the superior border of the symphysis [33]. Furthermore, we performed angle measurements according to the standardized procedure for the LCEA angle outlined in the literature, involving drawing a line connecting the center of the femoral head parallel to the longitudinal axis of the body, and another line connecting the center of the femoral head to the bony outer edge of the acetabulum [34].

All patients complained of hip symptoms, such as hip pain and discomfort, limited activity, and even lameness. All patients received at least 3 months of non-surgical treatment (physical therapy, anti-inflammatory drugs, etc.) before proceeding to surgery, and this non-surgical treatment failed.

### Surgical procedure

Under general anesthesia, the patient was placed in a supine position on a traction table equipped with a traction attachment. The lower limb was positioned on the operative side in 15° adduction, 15° internal rotation, and 15° forward flexion. The operative field was disinfected and draped. First, the fluoroscopic C-arm was used to establish the anterolateral portal to access the hip joint. A 17-gauge spinal needle was inserted to create the anterolateral portal, positioning the needle at approximately a 10° posterior angle and two-thirds the distance from the acetabulum to one-third the distance from the femoral head. Then a Nitinol wire was inserted through the spinal needle, confirming its direction toward the fovea using fluoroscopy. A 4.5-mm metal cannula was threaded over the wire, advancing it through the skin incision up to the hip joint capsule. The handle of the cannula was removed, followed by introducing an arthroscope to inspect the hip joint posteriorly and anteriorly, evaluating the articular cartilage, labrum, acetabular floor, synovium, and round ligament. Cartilage damage was assessed using the Outerbridge score, and any damage was repaired. The mid-anterior portal relative to the anterolateral viewing portal was established, proliferative and inflammatory synovial tissue was debrided, and the labral-acetabular junction was cleaned using a radiofrequency ablation device. The acetabular rim was polished using a 5.5 mm burr (Smith & Nephew), followed by reattaching the labrum to the acetabular rim by sequentially placing 2.3-mm biodegradable suture anchors single-loaded with high-strength permanent sutures (Smith & Nephew, Inc. Andover, MA, USA) to secure the detached labral tissue. The traction on the hip joint was released, and the hip joint was flexed to 35°- 40° to relax the anterior aspect of the capsule and access the peripheral compartment. We continued to debride proliferative and inflammatory synovial tissue to expose peripheral femoral cam lesions. Then, any labral contact was noted after flexing the hip. Using a 5.5-mm burr (Smith & Nephew) in the high-speed forward mode, the hip joint was further polished. Subsequently, we confirmed the absence of hip impingement post-flexion. After copiously irrigating the joint to remove bone shavings, the joint capsule was sutured using FiberWire sutures, completing the operation.

### Postoperative rehabilitation protocol

On the first postoperative day, the patient was allowed to walk on crutches and bear partial weight on the affected limb. Within one month after the operation, the patient was allowed to walk on crutches and gradually bear full weight within the tolerable range. Within two to three months after surgery, full weight-bearing and normal activities of the hip joint could be resumed. Patient were engaged in strength training, gradually improving muscle strength and stability around the hip joint, which allowed them to return to normal daily activities and even sports.

### Evaluation criteria

Preoperatively and at a minimum of one year postoperatively, the Visual Analogue Scale (VAS), modified Harris Hip Scores (mHHS), and Hip Outcome Score—Activities of Daily Living (HOS-ADL) and the International Hip Outcome Tool-12 (iHOT-12) were used to evaluate changes in patient’s pain, quality of life, and psychological impact before and after surgery [3, 35]. Statistical changes in the above-mentioned rating scale values before and after clinical intervention did not represent clinical benefits and perceived symptom improvement for patients. Minimum clinically important difference (MCID) and Patient acceptable symptomatic state (PASS) are important tools for evaluating clinical benefit [2, 3], and are rarely used in domestic clinical research. Therefore, we calculated MCID (mHHS), MCID (HOS-ADL), PASS (mHHS) and PASS (HOS-ADL) based on mHHS and HOS-ADL scores to evaluate the postoperative clinical efficacy [3, 22]. We used published PASS cutoffs of 87 for the HOS-ADL and 74 for the mHHS [3, 36]. Additionally, we used published MCID values of 8 for the mHHS and 9 for the HOS-ADL [3, 37, 38].

### Statistical methods

SPSS20.0 statistical software (SPSS, USA) was used for analysis. Measurement data subject to a normal distribution (age, body mass index, follow-up time, pre- and post-operative scores and their change values, and imaging angles) were expressed as χ ± s, and an independent sample test was used for comparison between groups. Preoperative and postoperative VAS, mHHS, HOS-ADL, and iHOT-12 scores were compared using paired test. Count data were expressed as frequencies (%). The gender of the two groups was analyzed using the chi-square test, and the PASS and MCID of the two groups were analyzed using the Fisher exact probability method. The α value of the test level is 0.05 on both sides.

## Results

### Baseline characteristics and follow-up information

In this study, the mean duration of symptoms prior to treatment was 11.8 months (standard deviation ± 8.9 months). The follow-up period was at least one year, with an average duration of 21.4 months (standard deviation ± 8.2 months). Postoperative outcome scores in this study were collected at the last postoperative follow-up (at a minimum of one year postoperatively). Twenty-two patients completed follow-up and two patients were lost. Among these 22 patients, 7 patients were ≥ 40 years old while 15 patients were < 40 years old. There were ten men and 12 women. In this study, the comparison between patients aged ≥ 40 and ≤ 40-years revealed no statistically significant differences in preoperative characteristics such as gender, BMI, α angle, Tönnis angle, and LCEA (all *P* > 0.05, Table 1). Similarly, when comparing male and female groups, there were no statistically significant differences in preoperative age, BMI, α angle, Tönnis angle, and LCEA angle (all *P* > 0.05) (Table 2).

**Table 1:** Preoperative parameter analysis based on age (X ± S, scores)

Indicators	≥ 40(*n* = 7)	<40(*n* = 15)	t-Value	*P*-Value
Male	28.6%	53.3%	—	0.381
Female	71.4%	46.7%	—	0.381
BMI	24.2 ± 3.57	21.94 ± 3.38	1.433	0.167
α angle	62.55 ± 7.86	64.5 ± 8.63	-0.504	0.619
Tönnis angle	8.06 ± 2.46	4.95 ± 4.37	1.745	0.096
LCEA angle	23.4 ± 0.89	23.19 ± 1.32	0.396	0.697

**Table 2:** Preoperative parameter analysis based on gender (X ± S, scores)

Indicators	Female(*n* = 12)	Male(*n* = 10)	t-Value	*P*-Value
Age	37.08 ± 9.65	31.8 ± 9.04	1.315	0.203
BMI	22.44 ± 3.57	22.92 ± 3.64	-0.315	0.756
α angle	64.72 ± 7.97	62.86 ± 8.9	0.517	0.611
Tönnis angle	6.27 ± 5.17	5.54 ± 2.42	0.412	0.685
LCEA angle	23.11 ± 1.45	23.43 ± 0.82	-0.614	0.546

### Surgical situation

**Fig. 1:**
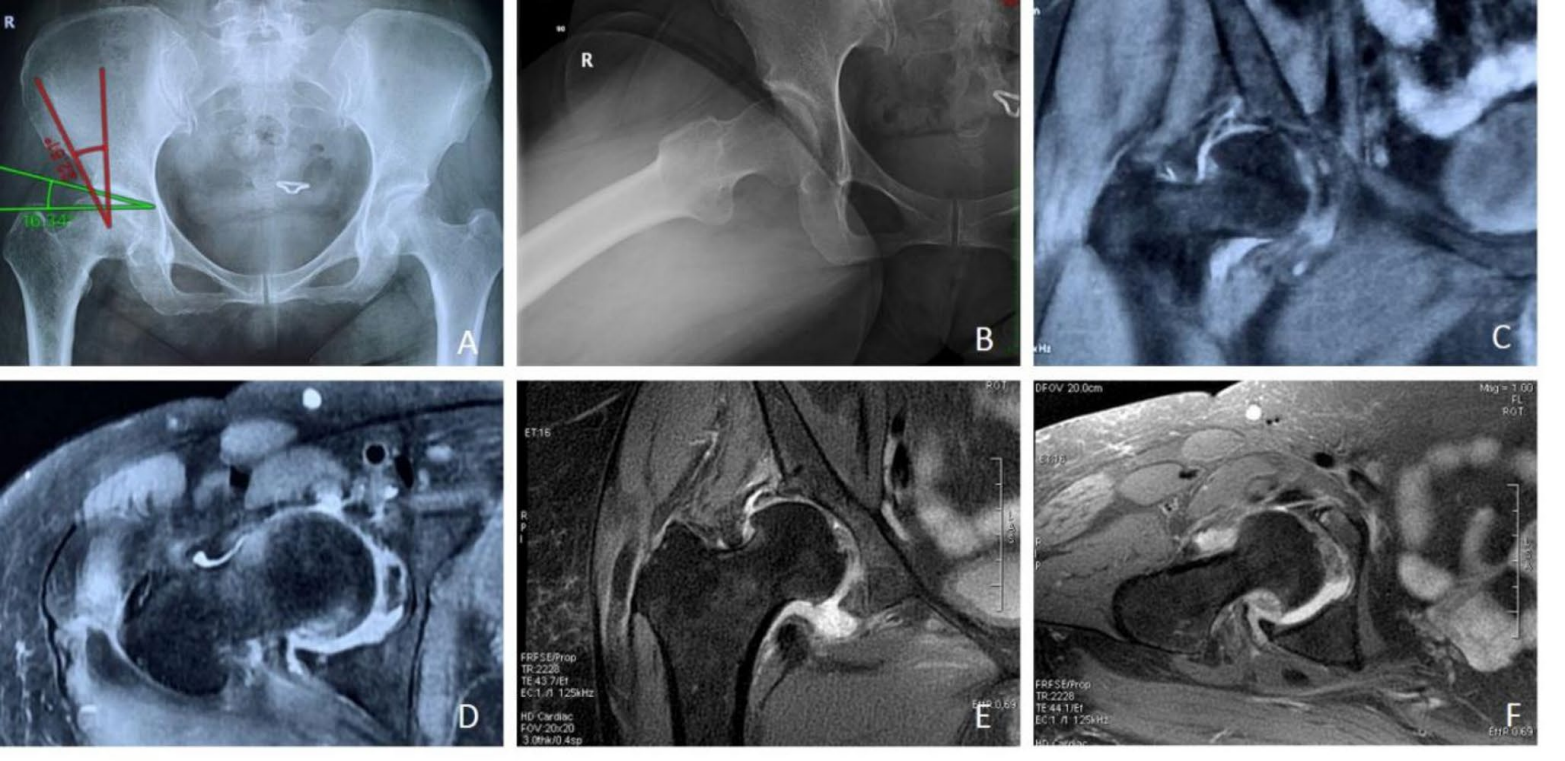
The patient was a 53-year-old female with right-sided borderline developmental dysplasia of the hip and cam impingement syndrome. Arthroscopic hip cartilage trimming, labral suturing, head and neck camplasty, and capsulorrhaphy were performed. (**A**) Preoperative pelvic anteroposterior X-ray shows critical dysplasia of the right hip joint, with an LCEA of 22.51° and a Tönnis angle of 16.34°. (**B**) Preoperative Dunn position X-ray shows cam deformity at the junction of the right femoral head and neck. (**C**) and (**D**) Preoperative MRI shows labral tear. (**E**) and (**F**) MRI 6 months after surgery shows healing of the labral tear

**Fig. 2:**
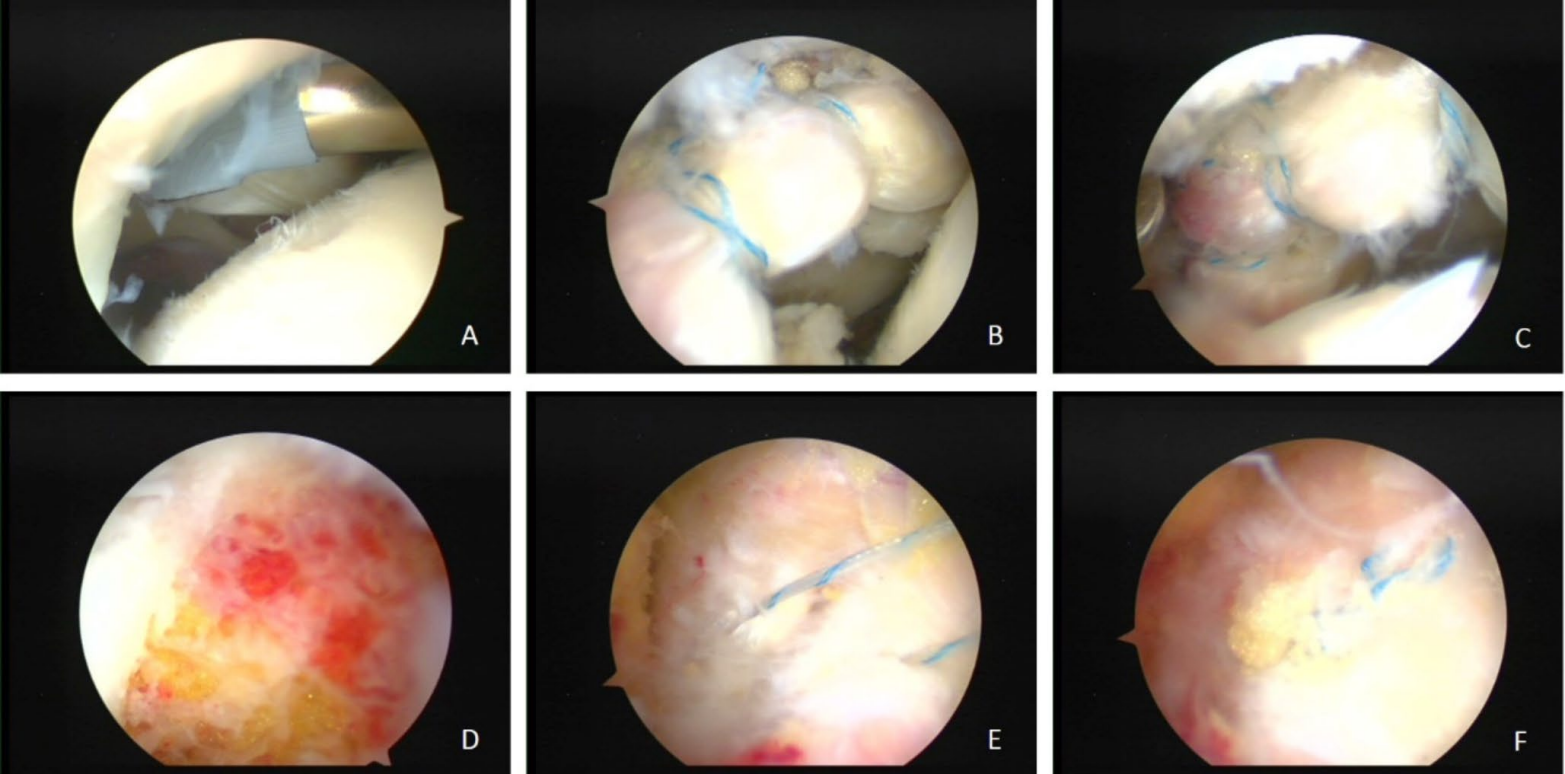
The patient was a 53-year-old female with right-sided borderline developmental dysplasia of the hip and cam impingement syndrome. Arthroscopic hip cartilage trimming, labral suturing, head and neck camplasty, and capsulorrhaphy were performed. (**A**) Cartilage degeneration. (**B**) and (**C**) Labral repairing. (**D**) Cam shaping at the femoral head-neck junction area. E .and F. The joint capsule was sutured

Intraoperative arthroscopy showed that all patients had varying degrees of labral damage. Nine cases also had acetabular cartilage damage (Outerbridge grade 1–2), and all patients had cam deformity at the junction of the femoral head and neck. All surgeries were successful. There was no important neurovascular injury during the operation, and the surgical incisions healed well after the operation. All patients did not undergo revision surgery during the follow-up (Figs. 1 and 2).

### Follow-up outcomes

Compared with pre-operation, the VAS score of the patient during postoperative follow-up was significantly lower (*P* < 0.001), and the mHHS score, HOS-ADL score and iHOT-12 score were significantly increased (*P* < 0.001). Postoperative MCID (mHHS) was 81.8%, MCID (HOS-ADL) was 77.3%; postoperative PASS (mHHS) was 86.4%, and PASS (HOS-ADL) was 72.7% (Tables 3 and 4).

**Table 3:** Comparison of pre- and postoperative pain and functional scores in patients with borderline developmental dysplasia of the hip undergoing arthroscopic surgery (X ± S, scores, 22 cases)

Indicators	Preoperative	Postoperative	t-Value	*P*-Value
VAS Score	5.27 ± 1.58	1.55 ± 1.1	9.05	<0.001
mHHS Score	64.84 ± 13.58	90.4 ± 10.11	-7.07	<0.001
HOS-ADL Score	68.92 ± 11.76	88.91 ± 9.51	-7.66	<0.001
iHOT-12 Score	49.32 ± 12.01	79.61 ± 15.89	-8.15	<0.001

Note: VAS, visual analogue scale; mHHS, modified Harris Hip Function Score; HOS-ADL, Hip Outcome Score-Daily Living Scale; iHOT-12, International Hip Outcome Scoring Tool

**Table 4:** Postoperative follow-up MCID and PASS values (cases, %)

Indicators	mHHS Score	HOS-ADL Score
MCID	18(81.8%)	17(77.3%)
PASS	19(86.4%)	16(72.7%)

Note: MCID: minimum clinically significant change value; PASS: patient acceptable symptom state

There were significant differences in the preoperative HOS-ADL score (*P* < 0.001), postoperative HOS-ADL score (*P* < 0.001), preoperative iHOT-12 score (*P* = 0.049) and postoperative iHOT-12 scores (*P* = 0.005) between the male and the female group. There was no significant difference in the preoperative VAS scores, postoperative VAS scores, preoperative mHHS scores, postoperative mHHS scores, MCID (mHHS), MCID (HOS-ADL), VAS score change value, mHHS score change value, HOS-ADL score change value and iHOT-12 score change value between these two groups (all *P* > 0.05) (Table 5).

**Table 5:** Preoperative and postoperative scores and postoperative satisfaction differences based on gender (X ± S, scores)

Indicators	Female	Male	t-Value	*P*-Value
Preoperative VAS Score	5.42 ± 2.02	5.1 ± 0.88	0.459	0.651
Preoperative mHHS Score	62.92 ± 15.9	67.14 ± 10.52	-0.718	0.481
Preoperative HOS-ADL Score	62.02 ± 9.36	77.2 ± 8.73	-3.906	<0.001
Preoperative iHOT-12 Score	44.77 ± 10.64	54.78 ± 11.72	-2.1	0.049
Postoperative VAS Score	1.83 ± 1.19	1.2 ± 0.92	1.371	0.185
Postoperative mHHS Score	86.71 ± 10.87	94.83 ± 7.35	-2.007	0.059
Postoperative HOS-ADL Score	83.22 ± 9.28	95.74 ± 3.23	-4.053	<0.001
Postoperative iHOT-12 Score	71.61 ± 16.65	89.21 ± 7.9	-3.25	0.005
MCID(mHHS)	66.7%(8)	100%(10)	—	0.096
MCID (HOS-ADL)	75%(9)	80%(8)	—	1.0
Change in VAS Score	-3.58 ± 2.39	-3.9 ± 1.29	0.375	0.712
Change in mHHS Score	23.79 ± 21.65	27.69 ± 9.48	-0.527	0.604
Change in HOS-ADL Score	21.2 ± 14.87	18.54 ± 8.67	0.498	0.624
Change in iHOT-12 Score	26.84 ± 20.87	34.43 ± 11.9	-1.017	0.321

Note: VAS: visual analogue scale; mHHS: modified Harris Hip Function Score; HOS-ADL: Hip Outcome Score-Daily Living Scale; iHOT-12: International Hip Outcome Scoring Tool. MCID: minimal clinically significant change

There was no statistical difference in the preoperative VAS score, postoperative VAS score, preoperative mHHS score, postoperative mHHS score, preoperative HOS-ADL score, postoperative HOS-ADL score, and preoperative iHOT-12 score, postoperative iHOT-12 score, MCID (mHHS), MCID (HOS-ADL), VAS score change value, mHHS score change value, HOS-ADL score change value and iHOT-12 score change value between patients aged ≥ 40 and ≤ 40 years (all *P* > 0.05, Table 6).

**Table 6:** Differences in preoperative and postoperative scores and postoperative satisfaction based on age (X ± S, scores, 22 cases)

Indicators	≥ 40	<40	t-Value	*P*-Value
Preoperative VAS Score	5.0 ± 1.15	5.4 ± 1.76	-0.544	0.592
Preoperative mHHS Score	67.01 ± 7.62	63.82 ± 15.76	0.505	0.619
Preoperative HOS-ADL Score	66.19 ± 7.91	70.19 ± 13.24	-0.736	0.470
Preoperative iHOT-12 Score	46.9 ± 7.55	50.45 ± 13.7	-0.636	0.532
Postoperative VAS Score	1.43 ± 1.13	1.6 ± 1.12	-0.332	0.743
Postoperative mHHS Score	85.39 ± 12.2	92.74 ± 8.44	-1.653	0.114
Postoperative HOS-ADL Score	85.09 ± 10.89	90.69 ± 8.6	-1.311	0.205
Postoperative iHOT-12 Score	71.91 ± 19.28	83.2 ± 13.26	-1.61	0.123
MCID (mHHS)	71.4%(5)	86.7%(13)	—	0.565
MCID (HOS-ADL)	71.4%(5)	80%(12)	—	1.0
Change in VAS Score	-3.57 ± 1.99	-3.8 ± 1.97	0.253	0.803
Change in mHHS Score	18.37 ± 14.58	28.92 ± 17.4	-1.388	0.18
Change in HOS-ADL Score	18.9 ± 10.66	20.5 ± 13.24	-0.279	0.783
Change in iHOT-12 Score	25.01 ± 18.07	32.75 ± 17.2	-0.968	0.344

Note: VAS: visual analogue scale; mHHS: modified Harris Hip Function Score; HOS-ADL: Hip Outcome Score-Daily Living Scale; iHOT-12: International Hip Outcome Scoring Tool. MCID: minimal clinically significant change

### Surgical complications

One female patient developed perineal numbness after the operation, which recovered > two months after the operation without any special treatment. All patients had no serious postoperative complications, such as wound infection, femoral neck fracture, sciatic nerve injury, femoral nerve injury, hip instability, or hip dislocation.

## Discussion

This study demonstrated that arthroscopic interventions including hip cartilage trimming, torn labral suture fixation, femoral camplasty, and capsulorrhaphy yield favourable clinical outcomes in patients with BDDH. Postoperatively, significant improvements were observed in VAS, mHHS, HOS-ADL, and iHOT-12 scores. Additionally, the high postoperative MCID and PASS values indicated good clinical efficacy.

Treatment options for BDDH encompass conservative management, arthroscopic surgery, and PAO [15]. Consensus on the optimal treatment regimen for BDDH is currently lacking. When selecting a surgical strategy for BDDH, ascertaining the stability and presence of impingement in the hip joint is crucial. Furthermore, the individual characteristics of each patient must be thoroughly evaluated. Both hip arthroscopy and PAO effectively alleviate the clinical symptoms of patients with BDDH [39]. Generally, patients with impingement as the main symptom should undergo hip arthroscopy, while patients with instability as the main symptom should undergo PAO. For patients with severe acetabular retroversion, reverse PAO represents a better option [15, 24, 40, 41]. McClincy et al. [19] followed up 39 BDDH patients who underwent PAO for at least two years and found that 94% of the patients had significant postoperative curative effects. Their mobility, quality of life, and overall health status were improved compared with those before surgery, as well as their hip joint symptoms.

PAO is a more invasive surgical procedure, whereas hip arthroscopy is generally perceived as more acceptable to patients due to its less invasive nature [16]. Multiple studies found that hip arthroscopy has better short and mid-term postoperative outcomes in patients with BDDH [2, 20–23]. Chandrasekaran et al. [2] followed up 55 BDDH patients for at least two years after hip arthroscopy and found that the patient’s postoperative HOS-ADL, HOS-SSS and other scores were improved compared with those before surgery, and the pain was significantly reduced. Furthermore, 83.6% of the patients achieved good or even excellent satisfaction scores. Moreover, hip arthroscopy shows good long-term postoperative efficacy in patients with BDDH [24]. In a study examining BDDH patients with femoroacetabular impingement who underwent hip arthroscopy, the follow-up averaged 9.6 years. Postoperatively, both group and control groups showed significant improvement in their scores compared to preoperative values. There was no significant difference in the MCID between the two groups [24]. Additionally, the revision rate in the BDDH group was significantly lower at 5.3% compared to 10.4% in the control group [24]. The author emphasized that preserving the labrum to the greatest extent during surgery and meticulously suturing the joint capsule are critical for achieving a favourable long-term prognosis [24]. Similarly, another long-term (≥ 10 years) clinical follow-up study obtained similar results [25]. This study found that hip arthroscopy can significantly improve the mHHS score, HOS-ADL score, HOS-SSS score and WOMAC score of BDDH patients with femoroacetabular impingement symptoms, with > 80% of patients reaching MCID [25]. Domb et al. [42] followed up BDDH patients who underwent hip arthroscopy for at least ten years and found that the overall survival rate after surgery was 82.2%, and the scores on all scales were significantly improved. The results of our study are similar to the above-mentioned research. We found that pain in patients with BDDH was significantly reduced after hip arthroscopy, and the mHHS score, HOS-ADL score and other scores were significantly improved compared with preoperative scores.

Clinical rating scales can be used to evaluate clinical surgical effects, and the results of the scales are usually interpreted in terms of statistical data (*P* value). However, statistical significance does not mean clinical significance; hence, one should not draw conclusion in clinical research based only on the *P* value. MCID usually refers to the smallest change in a clinical rating scale that patients perceive as beneficial [43]. MCID is a tool that links changes in scores to clinical outcomes. Thus, meeting or exceeding MCID values indicates important changes in clinical effectiveness. PASS represents the value of a certain rating scale, and exceeding this value indicates that the patient is satisfied with the therapeutic effect [44, 45]. Therefore, if the differences in various scales before and after treatment or between groups fail to meet the thresholds for MCID or PASS, they might not be clinically meaningful even if the results are statistically significant. In international clinical research, the early clinical effects of hip arthroscopy garnered considerable attention, extensively utilizing assessment tools such as MCID and PASS. However, they are rarely used in domestic clinical studies. Previous studies found that the MCID and PASS of patients with BDDH after hip surgery were 79.5-92.9% and 62.5-81%, respectively [3, 25, 35]. The results of our study are similar, with postoperative MCID (mHHS) of 81.8%, MCID (HOS-ADL) of 77.3%, PASS (mHHS) of 86.4%, and PASS (HOS-ADL) of 72.7%. Therefore, hip arthroscopy for BDDH has definite clinical effects and high patient satisfaction.

Although hip arthroscopy is effective in treating BDDH, the postoperative failure rate is still a problem that cannot be ignored. Usually, patients with BDDH who undergo repeated hip arthroscopy, as well as THA or PAO after hip arthroscopy are defined as failure of initial hip arthroscopy [46]. Previous studies indicated that the failure rate of initial hip arthroscopy for BDDH is 0-46%, the overall failure rate is 14.1%, and the average reoperation rate is 8.5%, of which the reoperation incidence for THA and PAO is 4.4-26.0% [1, 7, 8, 26] and 4% [26, 46], respectively. In this study, no patient underwent reoperation, which might be related to our strict control of surgical indications and standardized intraoperative procedures. We excluded patients with Tönnis grade ≥ 2, and all patients had minor acetabular cartilage damage (Outerbridge grade 1–2), the criteria that might effectively reduce the occurrence of reoperation, especially THA.

Various factors may lead to poor results of hip arthroscopy in BDDH treatment [1, 25, 42]. Studies found that preoperative tearing of the round ligament can lead to lower mental and psychological scores in BDDH patients after hip arthroscopy, making it more difficult to achieve PASS, and significantly increasing the patient’s risk of reoperation or even THA [1, 20]. Hatakeyama et al. [8] followed up BDDH patients who underwent hip surgery and found that the preoperative predictors of poor patient prognosis were age ≥ 42 years, Shenton line rupture, hip OA, preoperative Tönnis angle ≥ 15°, and VCA angle ≤ 17°. Intraoperative predictors of poor outcome included severe acetabular cartilage damage and even mild femoral cartilage damage. The authors recommend careful selection of hip surgery for BDDH patients with the above-mentioned risk factors. In a long-term follow-up study, more than half of BDDH patients (52.6%) were older than 40 years, and the rate of repeat THA surgery after hip arthroscopy was 24% [25]. The authors stated that risk factors for THA reoperation include older age, higher Tönnis grade (Tönnis grade ≥ 2), Outerbridge grade 4 cartilage lesions (and previous microfracture surgery), and Tönnis angle > 15° [25]. In this study, we observed that patients aged < 40 years demonstrated greater improvements in the rating scale and MCID values compared to those aged ≥ 40 years. However, the differences between the two age groups were not statistically significant. Beals et al. [25] also found that compared with older patients (≥ 40 years old), younger patients (< 40 years old) had a higher percentage of MCID and PASS, but there was no difference between the two groups. Given the limited sample size of this study, we plan to conduct a large-scale clinical follow-up study to further investigate the impact of age on reoperation rates in patients with BDDH. Additionally, our analysis of the gender effect on postoperative outcomes revealed no significant differences in score changes and MCID between men and women, suggesting that gender might not be a risk factor influencing postoperative outcomes.

This study has several limitations. First, the sample size was small. Second, the average follow-up was 21.4 ± 8.2 months, representing a short to mid-term period post-surgery. Thus, the long-term clinical effects remain undetermined. Therefore, expanding the sample size and extending the follow-up for more comprehensive future research are essential.
